# Self-esteem in new light: a qualitative study of experiences of internet-based cognitive behaviour therapy for low self-esteem in adolescents

**DOI:** 10.1186/s12888-023-05328-0

**Published:** 2023-11-07

**Authors:** Matilda Berg, Helena Klemetz, Tomas Lindegaard, Gerhard Andersson

**Affiliations:** 1 Department of Behavioural Sciences and Learning, Linköping University, Linköping, SE-58183 Sweden; 2 Psykologpartners, Private practice, Linköping, Sweden; 3 Department of Biomedical and Clinical Sciences, Linköping University, Linköping, Sweden; 4 Department of Clinical Neuroscience, Karolinska Institute, Stockholm, Sweden

**Keywords:** Self-esteem, Qualitative, Internet-based cognitive behaviour therapy, Thematic analysis, Self-compassion, Self-determination theory

## Abstract

**Background:**

Low self-esteem is common and can be impairing for adolescents. Treatments that primarily target low-esteem are lacking. Internet-delivered cognitive behaviour therapy (ICBT) is a treatment that can be used for adolescents but ICBT is yet to be evaluated for low self-esteem using qualitative methods. The aim of this study was to investigate experiences of participating in a novel ICBT treatment for adolescents suffering from low self-esteem.

**Method:**

Fifteen adolescent girls who had received ICBT consented to participate in a semi-structured qualitative telephone interview at post-treatment. Data were analysed and categorised using inductive Thematic Analysis.

**Results:**

Four overarching themes were identified; (1) Increased awareness and agency in difficult situations, (2) Enhanced self-image, (3) Unique but not alone, and (4) Widened understanding and new perspectives. Participants reported positive changes in their thinking and behaviour, as well as helpful learning experiences in relation to themselves and their self-esteem. For instance, participants described a more self-accepting attitude, learned how to manage negative thoughts, and experienced an increased sense of connection to others.

**Conclusion:**

The results suggest that ICBT is experienced as helpful and will inform further use and development of ICBT for low self-esteem. Future studies should validate and further evaluate experiences of ICBT for low self-esteem in other settings and in particular for boys as the study only include female participants.

**Supplementary Information:**

The online version contains supplementary material available at 10.1186/s12888-023-05328-0.

## Introduction

Low self-esteem is characterized by a lack of self-worth, negative self-evaluations and wishes for more self-respect [1, 2]. Various definitions of self-esteem exist, with one common definition defining self-esteem as a global evaluation of one’s self-worth [3]. Among adolescents, low self-esteem is associated with mental health problems, such as depression and anxiety, and it has been described as both a cause and consequence of such problems [1]. In addition, adolescence is a sensitive period during which young individuals undergo various physical and psychological changes that might be associated with low self-esteem. Problems with low self-esteem and negative self-evaluation are associated with various other problems, such as lower life satisfaction, problems with identity formation, and poorer physical health [4–6]. Thus, the close association between low self-esteem and mental health problems in adolescence suggests that it can be a relevant target for psychological treatment. There are different theoretical approaches to understanding low self-esteem such as a developmental [4] and cognitive perspective [3]. The present study was informed by a cognitive-behavioural theoretical approach focusing on how low self-esteem can be approached as a clinically relevant psychological problem [7].

Few studies have evaluated treatments of low self-esteem in adolescents. When studied, self-esteem has mostly been a secondary outcome measure in Cognitive Behavioural Therapy (CBT) for adolescents with depression [8]. For adults there are treatment studies on low self-esteem. These studies have mostly investigated CBT interventions [9, 10] based on the cognitive model by Fennell [7]. In this model self-esteem is defined as a schema, i.e. as a pervasive cognitive, emotional and physical way to react about one oneself, one’s self-worth and one’s relationships [9]. The treatment developed by Fennell mainly includes cognitive components, such as identifying and challenging negative self-evaluations and life-rules, as well as strategies to increase self-acceptance [7].

The treatment studies on low self-esteem in adults show promising results, but they have mostly been in a group format [10]. For adolescents, treatments effects have been smaller, possibly because self-esteem has not been the main treatment target [8].

Internet-based psychological treatment has the potential to increase access to treatment for the young and internet native population [11, 12]. Internet-based treatments usually contain psychoeducative texts and exercises, and can be delivered with therapist guidance via a secure platform [13]. They also have the potential to reduce costs and travel times, and can be more easily and rapidly be evaluated compared to standard face-to-face treatments [11], [14]. Partly because of this, the research support for Internet treatments, and in particular Internet-based CBT (ICBT) is growing, showing promising results also in youths [11, 12]. In light of these findings, ICBT could be a suitable treatment option for adolescents with low self-esteem. However, research on ICBT for low self-esteem among adolescents is also very limited, with exception of a few studies in which self-esteem has been measured as a secondary outcome [15, 16]. We recently conducted a pilot RCT of ICBT for adolescents with low self-with low self-esteem (N = 52) aged 15–19 years, which showed promising results [17]. Compared to the control group, the treatment lead to increases in self-esteem (between-group Cohen’s *d* = 1.18), quality of life (*d* = 0.80), and decreased symptoms of depression (*d* = 0.61) and anxiety (*d* = 0.69). The treatment modules had high user ratings in terms of perceived helpfulness and relevance in relation to the experienced problems [17]. The treatment was mainly inspired by the cognitive techniques developed by Fennell [7], but also self-determination theory [18] (SDT), and self-compassion theory [19]. Principles of SDT aim to foster improvements in self-esteem by increasing experienced autonomy, healthy relationships, and support a learning mindset in relation to achievements [18]. Self-compassion theory aims to foster a kind, friendly, non-judgemental relationship to oneself and potential set-backs, and can serve as an effective way to strengthen self-esteem [20]. SDT and self-compassion were integrated in the CBT treatment as many researchers and practitioners have identified the risks of only pursuing unstable, conditional foundations of self-esteem, such as extrinsic factors of success or admiration [3], [19]. Thus, SDT and self-compassion techniques describe how self-esteem can be strengthened in a healthy, sustainable way, helping young individuals to become more resilient in the presence of set-backs and life challenges.

While there are quantitative studies on the effects of treatment for low self-esteem, qualitative studies are more rare. Qualitative research methods can increase the understanding of treatment effects and how treatment content can be tailored and adjusted in relation to participants needs and wishes. Qualitative studies on ICBT for adults have mainly focused on whether ICBT can be a useful format for treatment delivery. Studies have reported mainly positive aspects such as flexibility, increased sense of autonomy and the value of support, but also feelings of stress in relation to working with the treatment material and a need of more personal guidance [21–23]. A few qualitative studies have been published on ICBT for adolescents, showing several positive aspects [24, 25]. For instance, Lenhard et al. [25] reported that guided ICBT increased self-efficacy and helpful normalization of symptoms. Berg et al. [24] found that treatment content could be remembered and used six months after treatment completion, but also reported differences in how explicitly and specifically the content was remembered. With these results in mind, Lenhard et al. [25] concluded that further qualitative research is needed to understand important process variables in ICBT for younger persons. Thus, more qualitative research is needed to better understand how participants perceive and engage in treatment programs, as well as how participants experience the process of change, in particular when researchers develop new treatments.

The present study was done in association with and following the RCT mentioned above [17]. We used qualitative method and telephone interviews with a smaller group of participants who had received the intervention. The aim was to evaluate how adolescents with low self-esteem experienced the ICBT program and it’s effects. More specifically, the ambition was to get a deeper understanding of the potential changes experienced by the participants during the course of treatment (both good and bad), how the treatment was perceived (in their own words), and to explore what they had learned and remembered about self-esteem when the treatment had ended.

## Method

The study was part of a controlled pilot trial [17] registered in clinicaltrials.org (NCT04737356). Participants were not financially compensated for participating in the treatment, completing assessments, or participating in the qualitative interview.

### Participants

The participants were a sub-sample from the treatment group in the pilot trial. Recruitment to the trial took place between January and February 2021, mainly through advertisements in social media, schools and mental health care units. Interested individuals could register on the study platform that included information about the study, the procedure, the staff and access to the full range of pre-treatment screening measures. Individuals who fulfilled the screening and met the inclusion criteria were contacted for a semi-structured telephone interview using Mini International Psychiatric Interview [26] (M.I.N.I). The information derived from the M.I.N.I interviews was used to guide inclusion or exclusion of participants. Adolescents with severe mental illness of any type were excluded, whether it was schizophrenia or severe forms of depression and anxiety. Decision on exclusion or inclusion was made by the research group including two licenced psychologists, a psychiatrist and four psychologist students in their final year of a five-year clinical psychology program. The principal investigator (GA) made the final decision on who to include or exclude.

The inclusion criteria included being 15–19 years old; experiencing clinically significant low self-esteem according to the Rosenberg Self-Esteem Scale (≤ 20 points) and the Robson Self-concept Questionnaire (≤ 120 point); having access to internet via computer or smartphone, ability to write and speak Swedish; not suffering from any severe psychiatric problems or suicidal ideation; if on medication having no changes in dosage during the last month and not participating in any interfering psychological treatment. For more information about inclusion procedure and treatment measures, see the trial report [17].

A total of 52 individuals were included in the original study. All participants in the treatment group (n = 26) were asked before starting treatment about their interest to participate in a qualitative interview at post treatment. Of these, 16 (61.5%) agreed to be contacted for the qualitative follow-up interview after treatment completion on a separate occasion. Thus, a strategic sampling was used. The participants had to give their informed consent to the study twice. First, they gave written consent in connection to the pre-treatment assessment, and then renewed their consent verbally by telephone in association with the qualitative interview. When contacted for the interview for the post-treatment assessment, one participant declined to participate due to lack of time.

A total of 15 (57.7%) interviews were conducted in March 2021. The participants had a mean age of 17.2 years (range 16–19) and all were girls. All participants opened 7 out of 7 (100%) of possible modules. More information about participant characteristics is presented in Table 1.

**Table 1:** Characteristics of participants ¹ The names in the Table are pseudonyms to secure the anonymity of the participants. [2]Age is presented in an approximate range to hide the exact age of the participants. ^3^*RSES* = Rosenberg Self-Esteem Scale. Scores under 15 indicates a low self-esteem

Participant [1]	Age [2]	Gender	RSES [3] pre treatment	RSES post treatment	Number of opened modules
Rut [3]	17–19	Female	11	9	7
Majken	17–19	Female	9	21	7
Sara	16–18	Female	3	19	7
Johanna	15–17	Female	5	13	7
Karin	17–19	Female	11	17	7
Mia	17–19	Female	10	13	7
Ebba	15–17	Female	11	17	7
Fia	16–18	Female	14	18	7
Lotta	16–18	Female	11	28	7
Freja	16–18	Female	14	20	7
Stina	16–18	Female	9	20	7
Tuva	16–18	Female	13	14	7
Amanda	16–18	Female	2	6	7
Aida	17–19	Female	11	18	7
Jenny	17–19	Female	9	15	7

### The ICBT program

The ICBT program included seven modules administered over seven weeks, and the participants received one module per week. The modules were mainly authored by the first author (MB), in collaboration with the third author (TL). The modules covered: (1) Introduction and rationale based on CBT and SDT, (2) Self-esteem related to experienced competence and cognitive restructuring, (3) Relationships and behavioural experiments, (4) Autonomy and how to increase self-assertiveness, (5) Self-compassion in the presence of setbacks, (6) Self-acceptance and allowing negative emotions, and finally (7) Maintenance plan focusing on how to prevent relapses. For more information see Berg et al. [17]. The treatment program was delivered via a secure platform requiring two-step authorisation [27]. All modules included psychoeducation and exercises. They were partly based on the cognitive model of Fenell, containing cognitive rationales and techniques such as cognitive restructuring, behavioural activation, and self-acceptance. Moreover, the modules were also informed by SDT that connects healthy self-esteem to the psychological needs of autonomy, healthy relationships and experienced competence. SDT thus suggest that individuals in order to gain a healthy self-esteem need to be able to influence their own life-decisions, have close relations and a focus on learning rather than avoiding failure. Further, one module contained psychoeducation and exercises on how to manage set-backs using self-compassion. Self-compassion has been suggested as an important factor for treatment success when treating adults and adolescents. The treatment was guided and each participants had their own therapist throughout the study. The role of the therapist was mainly to provide feedback on the module exercises and to answer questions when needed. Therapists gave feedback on the exercises once a week and answered questions connected to the treatment within 24 h (not during weekends). The therapist support also included to help participants tailor exercises to individual needs, provide positive reinforcement, or give emotional support in presence of experienced setbacks. All communication between participants and their therapists took place through written messages via the treatment platform [27].

### Procedure and qualitative method

The qualitative interviews were conducted following the post-treatment telephone call in which structured interview questions were asked [17]. Participants were informed about the procedure and that their names would not be presented and possible quotations masked to secure anonymity. One female psychologist (HK) conducted the interviews which lasted between 20 and 60 min per participant. The interviewer is an expert in the field of child psychiatry and CBT, giving advice to the authors (MB, TL) when writing the treatment material in terms of content and choice of exercises. She also provided some general guidance to the student therapists about treating youths during the treatment period. She had had no previous contact with the participants. All interviews were audio recorded via computer and later transcribed verbatim. During the interview notes were taken as memory support for the interviewer. The transcribed interviews were anonymized and were not shared with the participants.

An interview guide was prepared by the research team and included questions about general experiences of the treatment, what participants had experienced as more or less helpful, and questions about gained knowledge and perceived changes as a result of treatment. The selection of questions was informed by previous qualitative studies in our group, and also an interest in gaining more understanding of what adolescents actually learn in ICBT (and not only what changes in symptoms they might experience). The open-ended questions could lead to follow-up questions, but also skipping of questions in case they had already been commented on. Moreover, based on the responses of the participants, the order of questions was sometimes changed. The interview guide is available in Supplemental online material A.

The material was analysed using thematic analysis [28] (TA). We decided to use TA as we believed it would be a useful approach to identify themes and patterns in the data that would be important or interesting, and that the methodology described in the work by Braun and Clarke [28] would be feasible for the interview material we expected to get. We also had good experiences of using this approach in our previous work. Given the lack of previous research in the area, the analysis was made in an bottom-up way, using inductive TA [28]. The theoretical framework of the study was critical realism [29], which acknowledges an objective reality while also stressing that how we perceive the world is influenced by our experiences and individual perspectives. This leads to an epistemological position that knowledge is both a reflection of an objective world and also contextual, i.e. influenced by preconceived notions and social context.

Briefly, TA in the original description is used to identify and analyse meaningful themes across the written material, using structured steps [28]. We used a semantic approach as described by Braun and Clarke [28]. The initial steps are to become familiar with the data and to generate codes, i.e. statements or quotes that capture meaningful concepts in relation to the research question(s). Subsequently, themes are identified and reviewed in relation to the codes and the data set, then finally named and differentiated into sub-themes. During the process it is important assure that the identified themes are in line with the raw material. Two of the authors conducted the analysis (HK and MB). One had extensive experience of ICBT and the intervention itself (MB), whereas the other author had extensive experience and expertise in adolescent psychiatry and CBT. The transcriptions and subsequent initial coding in relation to the aims of the study were led by one of the authors (HK). The codes were reviewed and discussed together with the first author (MB), to ensure that they closely reflected the raw material. Further analysis when searching and building of themes was made together with the first author (MB). The analysis was reviewed, revised and approved by the third and fourth author (GA and TL).

## Results

### Thematic analysis

We identified four overarching themes representing experiences of ICBT for low self-esteem: (1) Increased awareness and agency in difficult situations, containing the two sub-themes *less reactive and more reflective* and *noticing and changing unhelpful thoughts*; (2) Enhanced self-image, containing the three subthemes *increased self-acceptance*, *befriending oneself*, and *being more than I perform*; (3) Unique but not alone, consisting of the two sub-themes *everyone feels bad sometimes* and *less comparison with others*; (4) Deepened understanding and new perspectives including the two sub-themes *widened understanding of myself* and *self-esteem in new light*. See Table 2 for an overview of the themes and sub-themes. See Table 3 for examples of the steps in the analytic process. The quotes presented for each theme below are chosen based on how informative they were. The names of the participants are pseudonyms to secure anonymity.

**Table 2:** Overview of themes and sub-themes

Theme	Sub-Theme
1. Increased awareness and agency in difficult situations	1.1 Less reactive and more reflective 1.2 Noticing and changing unhelpful thoughts
2. Enhanced self-image	2.1 Increased self-acceptance 2.2 Befriending oneself 2.3 Being more than I perform
3. Unique but not alone	3.1 Everyone feels bad sometimes 3.2 Less comparison with others
4. Widened understanding and new perspectives	4.1 Widened understanding of myself 4.2 Self-esteem in new light

**Table 3:** Examples of steps in the analytic process

Example of quote	Codes	Theme	Subtheme
*“[…] yeah I feel that has really changed, I have become much more relaxed and stopped thinking about everything I say.* ”	More relaxed, stopped overthinking, think differently	1. Increased awareness and agency in difficult situations	1.2 Noticing and changing unhelpful thoughts
*“It [life] goes up and down but you can still maintain a good relationship with yourself and a good tone, as well, like in your head, in the mind, towards yourself.“*	Self-support, good relationship with self, good tone with oneself	2. Enhanced self-image	2.2 Befriending oneself
*“It is human to feel bad sometimes, you cannot feel good all the time.”*	It is human to struggle, cannot always feel good	3. Unique but not alone	3.1 Everyone feels bad sometimes
*“A good self-esteem is not that you are super happy, but more that you can handle the less good days in a better way”*	Self-esteem not same as positive emotion, self-esteem is being able to handle bad days	4. Widened understanding and new perspectives	4.2 Self-esteem in new light

### Increased awareness and agency in difficult situations

The overarching theme of increased awareness and agency in difficult situations reflect how participants experienced an increased sense of control in distressing or overwhelming moments. Participants described that they identify and analyse their feelings and thoughts in the moment to a greater extent as a result of the treatment. They also felt that this new level of awareness enabled them to act consciously rather than impulsively, as well as changing unhelpful ways of thinking. Two sub-themes were identified, less reactive and more reflective, and noticing and changing negative thoughts.

#### Less reactive and more reflective

While working with the treatment content the participants began to analyse emotionally intense situations in a step-by-step manner. They described an increased tendency to ask themselves reflective questions about *what* they feel, *why* they feel it, and what they *need* when facing moments of distress and negative emotions. One girl described this change in this way:

*I have begun to reflect on why I feel the way I do, what it means. [.]. If I feel stressed about things happening, if there have been a lot of impressions and thoughts and feelings, I have tried to identify what they are, like, what does this mean, what does it come from, what can I do about it. I can think and recognize why I feel the way I do*.

[Lotta]

Participants also described how this new, less reactive way of managing their emotions resulted in positive effects in challenging situations, such as a greater sense of calm and clarity. One participant shared her experience below:

”*I get less frustrated if I feel bad, and instead, I try to analyse how I feel, and see if I can understand the cause. Instead of directly deciding like how terrible it is that I am angry right now, I think more like, what do I need here? I need to analyze instead of criticizing myself*”.

[Mia]

As illustrated by the quotes, the participants expressed that they were able to slow themselves down to a greater extent, experiencing an increased ability to analyse their emotional reactions rather than immediately acting upon them.

#### Noticing and changing unhelpful thoughts

In addition to a more reflective way of managing intense emotions, participants described an increased awareness and agency in terms of noticing and changing destructive thoughts. They shared their experiences on how the treatment had helped them to become aware of their general thought patterns and the negative impact these thought patterns had on their behaviour. Participants also described how they learned to observe negative thoughts with a greater distance, but also how they managed to change their way of thinking to some extent. This is how one girl described this change:

“*I have started to think like I should not sit here and question everything I intend to say to this person, it is my father, it is my best friend. I have to be able to be myself in some way. Instead of putting up a facade… yeah I feel that has really changed, I have become much more relaxed and stopped thinking about everything I say.* ”

[Karin]

The quote reflects how Karin had begun to notice unhelpful thoughts connected to her father, and how she had managed to actually stop this way of thinking and started to interpret situations in a new way. The theme thus captures participants overall experiences of being able to identify but also change their unhelpful thoughts patterns.

### Enhanced self-image

In addition to the experiences of increased awareness and agency in difficult situations, participants also described changes in self-image and more positive feelings towards themselves as individuals following the treatment. During treatment, participants experienced increases in self-respect and noticed a shift from a pessimistic self-view to more self-acceptance and positive self-regard. Enhanced self-image was categorised into three sub-themes: increased self-acceptance, befriending oneself, and being more than what I perform.

#### Increased self-acceptance

Increased self-acceptance was a recurring theme. As a result of the treatment, participants experienced it easier to accept themselves and their negative emotions, and expressed an increased willingness to experience feelings without judgement or a urgency to change them. For some, this resulted in positive affect, but most participants expressed how acceptance increased a sense of being more at peace also when experiencing negative emotional states.

One girl described her new level of acceptance towards herself and all of her emotions:

“*I also in this way accept that I am the person I am today… I let myself have feelings and I let myself have bad moments instead of questioning this, I accept that I am not perfect, my mood is not perfect and I have to let myself be disappointed or angry or sad. As much as I let myself be happy and excited*.”

[Karin]

Another girl explained how she had stopped pretending that everything is well, applying a more accepting approach to her feelings:

*It was this with accepting that I am sad, it has helped a lot because when I shut it all inside, then it becomes, I do not feel better by shutting down and pretending that everything is alright… in this way it feels better to kind of accept, and just feel it and then deal with it*.

[Stina]

The quotes show that self-acceptance increased for participants and became a new way to relate to themselves. Instead of questioning, pretending, or changing what they feel, they described a greater tendency to be more accepting towards their own experiences, allowing themselves to be as they are.

#### Befriending oneself

Another change in terms of enhanced self-image was captured in how participants expressed the importance of becoming a better friend to themselves. They described how they had begun to manage self-criticism by treating themselves in a more supportive way. Befriending oneself also included a capacity to manage their own mistakes or personal imperfections in a gentler manner.

One girl described this change below:

*“It* [life] *goes up and down but you can still maintain a good relationship with yourself and a good tone, as well, like in your head, in the mind, towards yourself*.“

[Sara]

Further, one participant described her increased willingness to treat herself better:

“*I think like I would never do this to my best friend. Like I would never treat someone else like this, so what gives me the right to treat myself like this, instead of treating myself like a close friend that I love and like. It has really given me a different view of myself and my own relationship to myself and how important it actually is*.”

[Karin]

The quotes illustrate how participants had begun to view and treat themselves differently, becoming more friendly and supportive, through both ups and downs. This was often described as a new way of relating to oneself, having a deep, positive impact on their self-image.

#### Being more than I perform

A further example of improved self-image was reflected in quotes showing how participants had begun to separate external success and achievements from their sense of self-worth. As a result, some participants expressed how failures had become a chance to learn rather than lowering one’s self-esteem. Participants did not say that external success or achievements had become unimportant or irrelevant, but rather stressed how their sense of self-worth had become less dependent on them.

One girl expressed her new perspective:

“*I do not want to say that I do not take mistakes as seriously, but they do not have to have as great an impact on how good I am as a person and how much I am worth.“* (Fia).

Another girl described how this had helped her enjoy challenges more:

“*I do not have as much pressure on me when I do things. I think it is more fun now. I can fail, the whole world will not perish*. ” (Maja).

As illustrated above, participants felt that the treatment had helped them separate external success from feelings of self-worth, thus being more than what they perform. This seems to have resulted in more joy and openness in the presence of failure and new challenges.

### Unique but not alone

Another theme that emerged from the interviews was the feeling of being unique but not alone. By reading the psychoeducative texts and fictive case examples in the modules, participants felt recognition, while at the same time understanding how problems with low self-esteem can be experienced and expressed differently for different people. The case examples increased a sense of connection with others, as well as a sense of uniqueness in their own situation. Two sub-themes were identified: everyone feels bad sometimes and making fewer comparisons with others.

#### Everyone feels bad sometimes

As a result of the treatment, participants described the helpfulness in knowing that everybody feels bad sometimes. Participants also expressed the realization that others can suffer without showing it, hiding their internal struggles and feelings. Thus, the treatment had a normalizing effect on their own negative emotions and increased a sense of being equal to others.

One participant described this below:

*I know now that it is okay to feel bad sometimes. It is human to feel bad sometimes, you cannot feel good all the time*.

[Maja]

Furthermore, participant Karin highlighted how the treatment normalized her own negative feelings and decreased her self-doubt:

*I always think that everyone else loves themselves so much, that they think that they are so good at everything and that I am different because I do not hold myself in such high regard, but that it is actually very normal that everyone may not have the best self-esteem… The appearance can be very deceiving. I have always thought I am alone in this … but it is actually not the case*.

[Karin]

The quotes reflect how participants felt less lonely following treatment when facing negative emotions and life-struggles. The increased sense of connection with others seemed to have a relieving effect on the participants, leaving them with a sense of being normal when feeling sad, stressed or angry.

#### Making fewer comparisons with others

Along with the realization that all people suffer sometimes, participants also described how they had begun to compare themselves less with others. Participants expressed insights around how every individual have their own ways of being unique, and that people have different histories, personalities, and expressions of mental illness.

One participant expressed her thoughts on how unhelpful it can be to compare oneself with others:

“*I do not compare myself as much. I see myself as my own person and it is not possible to compare with others, because that is exactly what we are: not the same*. ”

[Majken]

Another participant shared how her new recognition of her own and others uniqueness has helped her to become more allowing towards herself:

*I try to think more kindly, like, why should I know everything straight away, like why do I compare myself like that with everyone else? You know, I can know things that others do not know, for example. [.]. yeah, why should one compare oneself when all of us are so different?*

[Johanna]

As the quotes illustrate, participants had begun to reflect upon how comparison with others affects them and displayed an increased awareness about how people can be different. In one way, they described a paradox; we all feel bad sometimes, but we feel bad in different ways. We are equal in our suffering but can suffer differently. This paradox seemed to have a positive impact on the participants, resulting in an increased sense of both connection and uniqueness among the participants.

### Widened understanding and new perspectives

The theme of widened understanding and new perspectives reflects how participants expressed that the treatment provided them with new understandings and perspectives of themselves as persons and their self-esteem. By reading the material and having weekly contact with their assigned therapist, participants described how they got to learn about themselves through helpful concepts and given rationales. In contrast to theme 2, that describes positive, emotional changes related to self-image, this theme rather reflects general effects of the psychoeducation within the programme and how it helped participants gain valuable intellectual insights to reflect upon. The theme was divided into two sub-themes: greater understanding of oneself and self-esteem in new light.

#### Greater understanding of oneself

Participants described a greater understanding of themselves because of treatment. Throughout the treatment, new information and perspectives were presented in a way that gave participants insights and new knowledge. They enjoyed reading the texts, while also having time and opportunity to reflect upon the content to understand how it could be applied to themselves and their own life situations.

One participant shared her appreciation of reading and reflecting upon the treatment material:

*I have had a lot of time to think about myself, like engage in self-discovery, and reflect on myself more. It has been very good, I have started to pay attention to things…*.

[Lotta]

Sara described how she understood herself differently after participating in treatment:

*A lot depends on how you had it before and how it was when you grew up […]. you can perceive things differently as a result of your upbringing and the people you had around you … like that there is a reason behind it, you are not just you because you want to and so on, but there is a real reason for it*.

[Sara]

In summary, the treatment content and its reflective exercises helped participants to reflect and apply new theoretical knowledge on themselves, widening their understanding of themselves as individuals.

#### Self-esteem in new light

A majority of participants described new perspectives on self-esteem as a result of reading the texts and participating in the treatment. They expressed how their perception of low self-esteem shifted from something problematic and unchangeable, into something that was understandable and possible to change. This resulted in feelings of hope and increased agency. Participants also expressed a new understanding about self-esteem as something beyond momentary feelings of success or failure, which increased the sense of being in control of one’s life and overall well-being in a long-term perspective.

One girl explained her new perspective on self-esteem:

“*A good self-esteem is not that you are super happy, but more that you can handle the less good days in a better way, just because you feel negative feelings, it does not have to mean that you have low self-esteem, like, you can handle it* ”.

[Stina]

Another participant described how the treatment content gave her a more flexible attitude towards her own self-esteem:

“*It was a thing in the treatment material, that you can see it [self-esteem] as an opinion, that has really stayed with me because then I felt like it is possible to change your self-esteem, it is not carved in stone*”.

[Tuva]

The quotes illustrate how self-esteem is seen in a new light, now viewed as something changeable, independent from negative or positive feelings. Participants described self-esteem as something they have the power to change themselves. For some of the participants, these new ways of understanding self-esteem were mostly described on an intellectual level, thus indicating that these participants had gained helpful knowledge yet to be transformed into actual behavioural changes. However, these participants also expressed that the rationales and texts increased their sense of courage, making them feel more ready to change their behaviour in line with their values and according to their new insights.

## Discussion

This study is the first to our knowledge to investigate participants’ experiences of an ICBT program for low self-esteem among adolescents (15–19 years old). The results showed that for many of the participants the treatment resulted in positive changes in various cognitive, behavioural, and affective processes. The thematic analysis of participant interviews conducted immediately after the treatment resulted in four overarching themes.

In the first theme *Increased awareness and agency in difficult situations*, participants described an increased ability to slow down and analyse difficult thoughts and emotions, which made them more able to deal with challenging situations in a more constructive way. In the second theme *Enhanced self-image*, the participants reported ways of relating to themselves such as being more accepting and friendly towards themselves in the presence of set-backs, as well as separating their sense of self-worth from external achievements. In the third theme *Unique but not alone*, participants expressed a greater connection with others and fewer comparisons, reporting a greater understanding that a part of being human is to occasionally experience aversive emotional states. Finally, in the fourth theme *Widened understanding and new perspectives*, participants reported new perspectives and knowledge both in relation to themselves and in relation to the construct of self-esteem, and how they had gained helpful insights by reading and reflecting upon the treatment content.

Overall, many of the themes identified in this study are in line with other qualitative studies on ICBT. For example, the experience of getting new perspectives, knowledge and insights as described in theme 4, was also reported by Berg et al. [24], in a study on ICBT for adolescent depression. In that study, participants expressed how the treatment content resulted in positive learning experiences that could be remembered six months after treatment. Another example concerns the described impact of normalization of symptoms, such as low self-esteem in this study. This effect was found in a previous OCD study [25]. Further, both in the Lenhard et al. [25] study as well as in the present study, participants expressed appreciation of the case examples and psychoeducative texts, describing feelings of recognition and possibilities for change.

As reflected in both theme 1, 2, and 3, participants expressed that they had started to use and apply specific CBT rationales and techniques provided within the treatment modules, such as noticing and changing negative thoughts or applying self-acceptance. The ability to pinpoint and describe use of treatment-specific techniques was also found in our previous qualitative study on adolescent depression [24], and similar findings have been reported in qualitative studies on adults participating in ICBT. For instance, Rozental et al. [21] reported how clients could describe the usage of specific key components in their ICBT program on perfectionism, such as behavioural experiments or activity scheduling.

However, some of the findings in the present study have to our knowledge not been specifically reported in other qualitative studies on ICBT for adolescents. This mainly concerns findings within the themes 2 and 3, in which participants reported becoming more friendly and allowing towards themselves, both as unique individuals and in the presence of failures or set-backs. The participants also described a heightened sense of connection with others when feeling sad or experiencing aversive emotional states. These findings are in line with self-compassion theory, which was a key component of the ICBT program tested. Self-compassion theory focuses on how to become more kind and non-judgemental towards oneself in the presence of challenges and feelings of inadequacy [19]. The experienced sense of connection with others that was expressed by the participants can be understood in the light of the theoretical concept of shared humanity in self-compassion theory. It stresses the importance of recognising that the experiences of suffering and personal inadequacy are universal human experiences [19]. From the perspective of self-compassion theory this recognition is helpful as it can connect us with humanity as a whole when suffering, rather than feeling isolated and alone. Given that self-compassion to our knowledge has not previously been incorporated in ICBT programs for adolescents, the lack of similar themes in other studies is understandable. However, since the participants highlighted these aspects as helpful, and given the potential connection between self-compassion and strengthened self-esteem [20], self-compassion might be of importance when constructing future interventions for adolescents with mental health issues.

Further, in themes 2 and 3 participants described various positive changes in their self-esteem in relation to their own achievements, relationships and self-image, such as less focus on external success to feel worthy, less comparison and more understanding in relation to others, and a strengthened trust in oneself as a unique individual. These aspects are in line with SDT [18], another key component of the program, that stresses the importance of fostering a learning mindset, the ability to engage in healthy relationships, as well as increased independence, in order to strengthen self-esteem in a sustainable way. The findings suggest that SDT might be a useful theoretical framework in future interventions of low self-esteem in adolescents, given the correspondence between the participants’ statements and the key components of SDT.

In addition, the qualitative findings in this study are in line with the quantitative results of the trial [17]. Together, the findings indicate that ICBT can successfully help adolescents to strengthen their low self-esteem. These positive results are also in line with meta-analyses on other mental health problems among adolescents, such as depression and anxiety [11, 12, 30]. Further, the results can be seen as a complement to the quantitative results [17], suggesting potentially active components in the ICBT program. Both the components of Fenells’ cognitive behavioural model of low self-esteem, such as noticing and challenging unhelpful negative self-evaluations, and the fostering of a more friendly, allowing attitude towards oneself in presence of set-backs using self-compassion theory, as well as the focus on healthy learning attitudes, relationships and expressed autonomy within SDT seems to be of value when helping adolescents strengthen their self-esteem.

Further, the results also highlight the potential advantage of targeting self-esteem as a primary focus in ICBT for adolescents. The value of primarily addressing transdiagnostic problems rather than specific diagnoses within the field of ICBT has been suggested by other studies. For example, ICBT have shown positive results in adults suffering from loneliness [31] and perfectionism [32]. Also, given the possible role transdiagnostic factors such as of low self-esteem have in the aetiology and maintenance of several disorders, it is possible that targeting low self-esteem directly can, at least in some cases, be more efficient than using multiple treatment protocols to address comorbidity [33].

Taken together, this study is in line with previous qualitative research on ICBT, suggesting that CBT delivered in an internet format can be perceived as helpful and useful, and that the content can be understood in line with the intended active mechanisms. Qualitative research on ICBT for adolescents is still limited. More studies using both qualitative and quantitative methods are needed [25], 30] in order to more fully understand the short- and long-term effects of the treatment, as well as the active mechanisms underlying the treatment effects and how the treatment is actually experienced.

### Limitations and reflexivity

The possibility to generalize the results is limited and is not typically a goal of qualitative research. The purpose of qualitative research is rather to identify themes that can be recognized and potentially transferable to other settings [34].

There are limitations and we did not for example get information about negative effects [35], which could be a result of the fact that participants had to agree to participate in the qualitative interview and that this perhaps was less likely to happen if they did not have positive expectations about the outcome of the ICBT intervention. The lack of information about negative effects could also be a consequence of the fact that only adolescents with a high adherence rate participated in the interviews. Adolescents who discontinued ICBT might have had other experiences with ICBT that have not been illuminated in this study. Including a more diverse group of participants might have nuanced the results and allowed for another range of positive and negative reports. However, we asked all participants about participation in the interview before treatment initiation and they thus agreed to participate before knowing how they would experience the treatment. Notably, the participants were only girls, which is in line with the reported preponderance of women in ICBT studies on adolescents [15, 36]. This pinpoints the future challenge of recruiting young men in ICBT studies.

In terms of reflexivity in TA [37], the two authors who analysed the treatment material were clinical psychologists with previous experience of treating adolescents with CBT or ICBT. It is likely that their previous knowledge and preconceptions about CBT affected their interpretation of transcriptions but also how the interviews were conducted. Moreover, two of the authors had developed the treatment material and could therefore have an interest in showing that it was appreciated. However, knowledge about a topic and even an interest to help adolescents with poor self-esteem can be a resource, since at least some knowledge about the treatment content is needed to analyse and understand how it has been perceived by the participants in relation to CBT theory and its intended active mechanisms. In addition, the program had been tested in a pilot trial and therefore we as researchers welcomed suggestions and comments for our future work.

## Conclusions

In conclusion, the present study investigated experiences of adolescents who had participated in an ICBT program targeting low self-esteem. Positive Enhancements in cognitive processes and behavior, as well as new perspectives on self-esteem, were stated as a result of having received the treatment. Our findings are similar to those found in previous studies on both adolescent and adult ICBT participants, for example regarding the helpful effects of applying various CBT techniques and the normalizing effect of the educational components of the programs. Some novel aspects were identified such as the recognition that suffering is a universal human experience and the importance of adopting a learning mindset, consistent with self-compassion theory and self-determination theory which informed the treatment alongside CBT. The qualitative results also points to several potential active mechanisms in the treatment although more research using both quantitative and qualitative methodologies is needed. Finally, the results also provide a complementary perspective and are in line with the positive quantitative results found in the original trial.

### Electronic supplementary material

Below is the link to the electronic supplementary material.

Supplementary Material 1: Interview guide

## Data Availability

The quotations in the present study are based on transcribed interviews which contain sensitive information, the data can therefore not be made publicly available. For any queries, please contact Tomas Lindegaard tomas.lindegaard@liu.se.

## References

[1] Ngo H, VanderLaan DP, Aitken M (2020). Self-esteem, symptom severity, and treatment response in adolescents with internalizing problems. J Affect Disord.

[2] Rosenberg M. Society and the adolescent self-image. Princeton University Press; 1965.

[3] Baumeister RF, Campbell JD, Krueger JI, Vohs KD (2003). Does High Self-Esteem cause better performance, interpersonal success, happiness, or healthier lifestyles?. Psychol Sci Public Interest J Am Psychol Soc.

[4] Moksnes UK, Espnes GA (2013). Self-esteem and life satisfaction in adolescents-gender and age as potential moderators. Qual Life Res Int J Qual Life Asp Treat Care Rehabil.

[5] Neff KD, McGehee P (2010). Self-compassion and psychological resilience among adolescents and young adults. Self Identity.

[6] Arsandaux J, Galéra C, Salamon R (2021). The association of self-esteem and psychosocial outcomes in young adults: a 10-year prospective study. Child Adolesc Ment Health.

[7] Fennell M. Overcoming low self-esteem (2nd Edition): a self-help guide using cognitive behavioural techniques. Brown Book Group; 2016.

[8] Taylor TL, Montgomery P. Can cognitive-behavioral therapy increase self-esteem among depressed adolescents: a systematic review. In: *Database of Abstracts of Reviews of Effects (DARE): Quality-Assessed Reviews [Internet]*. Centre for Reviews and Dissemination (UK); 2007. Accessed June 7, 2023. https://www.ncbi.nlm.nih.gov/books/NBK74020/.

[9] Waite P, McManus F, Shafran R (2012). Cognitive behaviour therapy for low self-esteem: a preliminary randomized controlled trial in a primary care setting. J Behav Ther Exp Psychiatry.

[10] Kolubinski DC, Frings D, Nikčević AV, Lawrence JA, Spada MM (2018). A systematic review and meta-analysis of CBT interventions based on the Fennell model of low self-esteem. Psychiatry Res.

[11] Ebert DD, Zarski AC, Christensen H (2015). Internet and computer-based cognitive behavioral therapy for anxiety and depression in youth: a meta-analysis of randomized controlled outcome trials. PLoS ONE.

[12] Vigerland S, Lenhard F, Bonnert M (2016). Internet-delivered cognitive behavior therapy for children and adolescents: a systematic review and meta-analysis. Clin Psychol Rev.

[13] Andersson G (2016). Internet-delivered psychological treatments. Annu Rev Clin Psychol.

[14] Andersson G, Titov N, Dear BF, Rozental A, Carlbring P (2019). Internet-delivered psychological treatments: from innovation to implementation. World Psychiatry.

[15] Berg M, Rozental A, de Brun Mangs J (2020). The role of Learning Support and Chat-Sessions in guided internet-based cognitive behavioral therapy for adolescents with anxiety: a Factorial Design Study. Front Psychiatry.

[16] Topooco N, Berg M, Johansson S (2018). Chat- and internet-based cognitive-behavioural therapy in treatment of adolescent depression: randomised controlled trial. BJPsych Open.

[17] Berg M, Lindegaard T, Flygare A (2022). Internet-based CBT for adolescents with low self-esteem: a pilot randomized controlled trial. Cogn Behav Ther.

[18] Crocker J, Park LE (2004). The costly pursuit of self-esteem. Psychol Bull.

[19] Neff K. Self-Compassion: the Proven Power of being kind to yourself. William Morrow; 2015.

[20] Thomason S, Moghaddam N (2021). Compassion-focused therapies for self-esteem: a systematic review and meta-analysis. Psychol Psychother.

[21] Rozental A, Kothari R, Wade T (2020). Reconsidering perfect: a qualitative study of the experiences of internet-based cognitive behaviour therapy for perfectionism. Behav Cogn Psychother.

[22] Lindegaard T, Kashoush F, Holm S, Halaj A, Berg M, Andersson G. Experiences of internet-based cognitive behavioural therapy for depression and anxiety among arabic-speaking individuals in Sweden: a qualitative study. BMC Psychiatry. 2021;21. 10.1186/s12888-021-03297-w. 10.1186/s12888-021-03297-w PMC8173836 34082745

[23] Patel S, Akhtar A, Malins S (2020). The acceptability and Usability of Digital Health Interventions for adults with Depression, anxiety, and Somatoform disorders: qualitative systematic review and Meta-synthesis. J Med Internet Res.

[24] Berg M, Malmquist A, Rozental A, Topooco N, Andersson G (2020). Knowledge gain and usage of knowledge learned during internet-based CBT treatment for adolescent depression - a qualitative study. BMC Psychiatry.

[25] Lenhard F, Vigerland S, Engberg H, Hallberg A, Thermaenius H, Serlachius E. On my own, but not alone - adolescents’ experiences of Internet-delivered cognitive behavior therapy for obsessive-compulsive disorder. PLoS ONE. 2016;11(10). 10.1371/journal.pone.0164311. 10.1371/journal.pone.0164311 PMC5053512 27711249

[26] Sheehan D, Lecrubier Y, Harnett Sheehan K (1997). The validity of the Mini International Neuropsychiatric interview (MINI) according to the SCID-P and its reliability. Eur Psychiatry.

[27] Vlaescu G, Alasjö A, Miloff A, Carlbring P, Andersson G (2016). Features and functionality of the Iterapi platform for internet-based psychological treatment. Internet Interv.

[28] Braun V, Clarke V (2006). Using thematic analysis in psychology. Qual Res Psychol.

[29] Willig C. Beyond appearances: a critical realist approach to social constructionism. Social Constructionist psychology: a critical analysis of theory and practice. Open University Press; 1999. pp. 37–51.

[30] Christ C, Schouten MJ, Blankers M, et al. Internet and computer-based cognitive behavioral therapy for anxiety and depression in adolescents and young adults: systematic review and Meta-analysis. J Med Internet Res. 2020;22(9). 10.2196/17831. 10.2196/17831 PMC7547394 32673212

[31] Käll A, Bäck M, Welin C (2021). Therapist-guided internet-based treatments for loneliness: a Randomized Controlled three-arm trial comparing cognitive behavioral therapy and interpersonal psychotherapy. Psychother Psychosom.

[32] Shafran R, Wade TD, Egan SJ (2017). Is the devil in the detail? A randomised controlled trial of guided internet-based CBT for perfectionism. Behav Res Ther.

[33] McManus F, Waite P, Shafran R (2009). Cognitive-behavior therapy for low Self-Esteem: a Case Example. Cogn Behav Pract.

[34] Larsson S (2009). A pluralist view of generalization in qualitative research. Int J Res Method Educ.

[35] Rozental A, Boettcher J, Andersson G, Schmidt B, Carlbring P (2015). Negative effects of internet interventions: a qualitative content analysis of patients’ experiences with treatments delivered online. Cogn Behav Ther.

[36] Topooco N, Byléhn S, Dahlström Nysäter E (2019). Evaluating the efficacy of internet-delivered cognitive behavioral therapy blended with synchronous Chat Sessions to treat adolescent depression: Randomized Controlled Trial. J Med Internet Res.

[37] Braun V, Clarke V (2019). Reflecting on reflexive thematic analysis. Qual Res Sport Exerc Health.

