# Supplementary material for: Self-esteem in new light: a qualitative study of experiences of internet-based cognitive behaviour therapy for low self-esteem in adolescents
Source: BMC Psychiatry. 2023 Nov 7;23:810. doi: 10.1186/s12888-023-05328-0 (PMC10631070; doi:10.1186/s12888-023-05328-0)
Supplement: Supplementary file 1 — Supplementary Material 1: Interview guide [file 12888_2023_5328_MOESM1_ESM.docx]

**Supplemental online material A**

**General questions about experiences of treatment**

What is your general experience of the treatment?

What was it about the treatment that has been extra helpful for you?

What was it about the treatment that you think should have been different?

**Questions about learning and perceived change from the treatment**:

Did you learn something from the treatment? If, what did you learn?

Is there something that has been extra important or meaningful for you to learn more about?

Have you used the knowledge in any way, in your everyday life? If so, how?

Have you noticed that you think, feel or do differently since the therapy began?

Do you think there was something in the treatment itself that led to those changes?

If you were to describe to a friend what you got from this treatment, what would you say?

Have you experienced any negative effects from the treatment? If so, which ones?

**If needed, ask more specified questions such as:**

Has the treatment changed anything about how you do, think or feel in relationships? If so, in what way?

Has the treatment changed anything about how you do, think or feel in relation to your own achievements? If so, in what way?

Has the treatment changed anything about how you do, think or feel about yourself and your own self-worth? If so, in what way?

Has the treatment changed anything about how you set boundaries and take care of your own needs? If so, in what way?

Has the treatment changed anything in how treat yourself in difficult times? If so, in what way?

**Last question**

Is there anything we missed asking you about that you want us to know?
